# Impact of coronary artery disease on systolic function in severe aortic stenosis—a multimodality imaging study

**DOI:** 10.1093/ehjimp/qyag095

**Published:** 2026-05-19

**Authors:** Reetta Hälvä, Satu Vaara, Suvi Syväranta, Miia Holmström, Johanna Federico, Touko Kaasalainen, Juha Peltonen, Satu Suihko, Jyri Lommi, Heikki Niemi, Valtteri Uusitalo

**Affiliations:** Radiology, HUS Diagnostic Center, Helsinki University Hospital and University of Helsinki, Helsinki, Finland; Radiology, HUS Diagnostic Center, Helsinki University Hospital and University of Helsinki, Helsinki, Finland; Radiology, HUS Diagnostic Center, Helsinki University Hospital and University of Helsinki, Helsinki, Finland; Radiology, HUS Diagnostic Center, Helsinki University Hospital and University of Helsinki, Helsinki, Finland; Radiology, HUS Diagnostic Center, Helsinki University Hospital and University of Helsinki, Helsinki, Finland; Radiology, HUS Diagnostic Center, Helsinki University Hospital and University of Helsinki, Helsinki, Finland; Radiology, HUS Diagnostic Center, Helsinki University Hospital and University of Helsinki, Helsinki, Finland; Heart and Lung Center, Helsinki University Hospital and University of Helsinki, Helsinki, Finland; Heart and Lung Center, Helsinki University Hospital and University of Helsinki, Helsinki, Finland; Heart and Lung Center, Helsinki University Hospital and University of Helsinki, Helsinki, Finland; Clinical Physiology and Nuclear Medicine, Helsinki University Hospital and University of Helsinki, Stenbäckinkatu 11, 00290 Helsinki, Finland

## Abstract

**Aims:**

The aim of this study was to determine the impact of obstructive coronary artery disease (CAD) and diffuse atherosclerosis on left ventricular systolic function, measured as global longitudinal strain (GLS) by cardiac magnetic resonance (CMR) feature tracking, in patients with severe aortic stenosis (AS).

**Methods and results:**

In this single-centre prospective cohort study, patients with severe AS and transcatheter aortic valve implantation (TAVI) between October 2018 and February 2022 were referred for transthoracic echocardiography, CMR, and TAVI computed tomography. Invasive coronary angiography was performed before TAVI. Ninety-four patients (80 ± 7 years) were included. The prevalence of obstructive CAD was 20 (21%) patients. The mean GLS in patients with severe AS was −24.0 ± 6.1%, and the median coronary calcium score was 802 (interquartile range 302–2130). GLS was not reduced in patients with obstructive CAD compared to other patients (23.7 ± 5.9% vs. 24.1 ± 6.2%, *P* = 0.83). A high coronary calcium score was not associated with GLS. Indexed left ventricular mass was independently related to GLS. Patients with an ischaemic scar on CMR had lower GLS (20.3 ± 5.1% vs. 24.6 ± 6.1%, *P* = 0.02).

**Conclusion:**

GLS correlated with left ventricular mass and the presence of ischaemic scar, suggesting that intrinsic myocardial disease, rather than concomitant CAD alone, accounts for reduced GLS in this population.

## Graphical Abstract

**Graphical Abstract:**
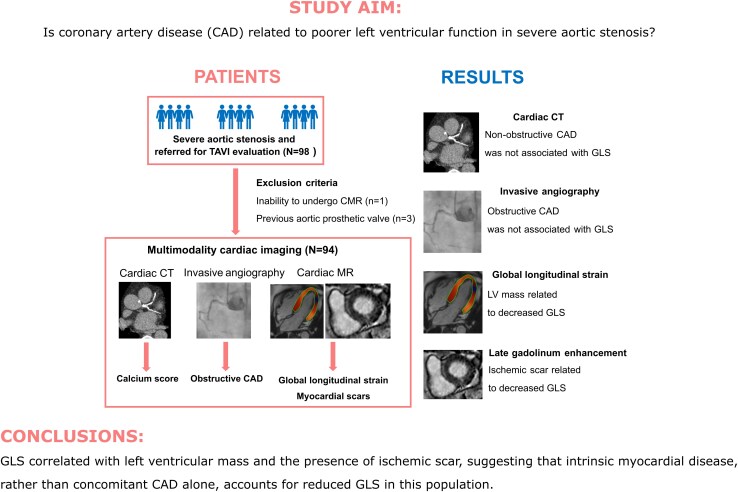
Summary of the study protocol and the main multimodality imaging results. CAD = coronary artery disease, CMR = cardiac magnetic resonance, CT = computed tomography, GLS = global longitudinal strain, TAVI = transcatheter aortic valve implantation.

## Introduction

In aortic stenosis (AS), elevated afterload eventually leads to left ventricular (LV) hypertrophy (LVH) and fibrosis.^1–3^ Optimally timed valve replacement can prevent irreversible myocardial remodelling, but the intervention criteria in patients with severe asymptomatic AS are debated.^1,4,5^ In recent years, transcatheter aortic valve implantation (TAVI) has emerged as an alternative to open-heart surgery, enabling valve replacement with lower perioperative risk.^1^ Depressed LV ejection fraction (EF) is a Class I indication in the European Society of Cardiology’s guideline for valve replacement if other causes for systolic dysfunction are excluded.^1^ However, low EF is a late presentation of myocardial disease, which might therefore limit its usefulness in achieving a good ventricular reverse remodelling outcome.^6^

Global longitudinal strain (GLS) is an earlier marker of cardiac dysfunction than EF.^7^ It has shown prognostic value even at moderate AS.^8^ Therefore, GLS is a promising method for guiding early valve replacement in AS. However, GLS might be influenced by extravalvular comorbidities such as coronary artery disease (CAD), which is common in AS patients.^9,10^ The combined severe AS and decreased coronary flow pressure in CAD may have an additive impact on the LV myocardial function. Moreover, diffuse atherosclerosis, endothelial dysfunction, and microvascular disease might accelerate the irreversible myocardial remodelling in severe AS and attenuate the long-term benefits of aortic valve (AV) interventions.

The advantage of cardiac magnetic resonance (CMR) imaging over echocardiography is that it is not dependent on a good acoustic window for GLS measurement. In addition, CMR is the reference standard for ventricular volumetry and scar imaging. In this prospective multimodality study, we evaluated the significance of obstructive CAD and diffuse atherosclerosis on LV systolic function measured as GLS by CMR feature tracking in patients with severe AS.

## Methods

### Study population

This study complies with the Declaration of Helsinki, and it was approved by the Ethics Committee of Helsinki University Central Hospital (HUS/1743/2018 and HUS/2471/2019) and the institutional research board (HUS46/2018). All the patients gave written informed consent.

**Figure 1:**
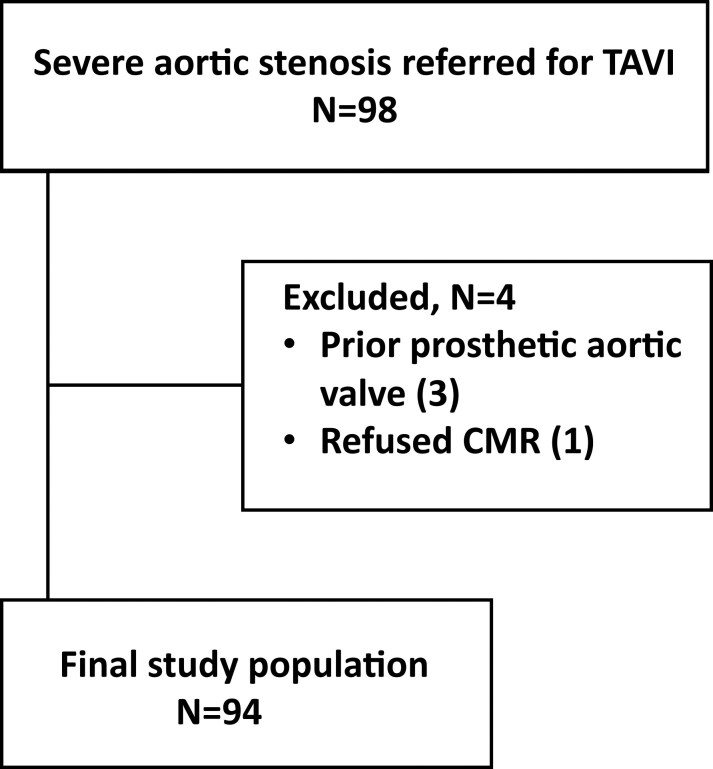
Flowchart of the study population.

**Figure 2:**
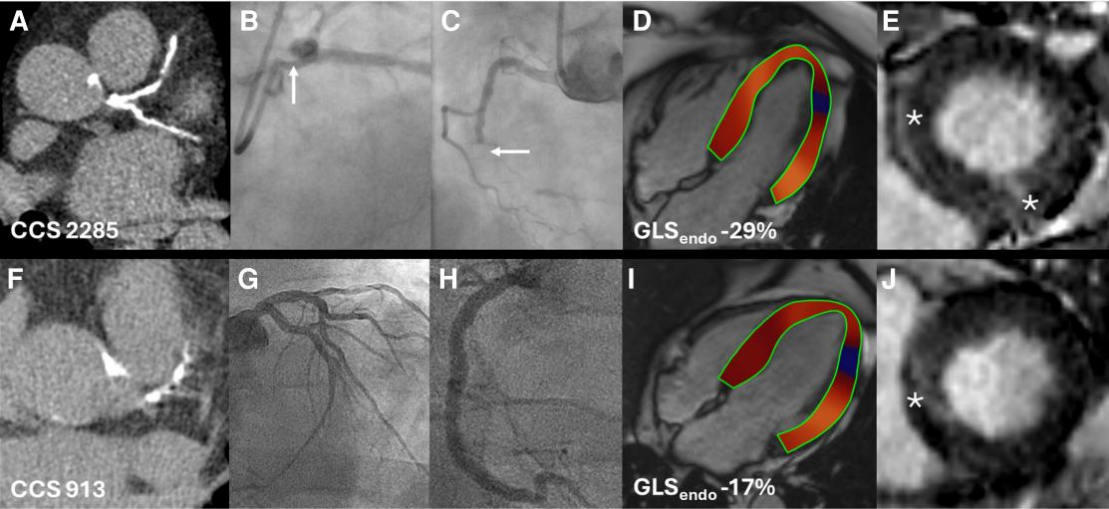
Multimodality imaging evaluation of severe aortic stenosis (AS) in our study. A patient with severe coronary artery disease (CAD) and AS (top row) and a patient with non-obstructive atherosclerosis and AS (lower row). Atherosclerotic burden was measured by the coronary calcium score (CCS) (A and F). Invasive coronary angiography was done to assess coronary stenoses (B, C, G, and H). Left main stenosis (B) and chronic total occlusion (C) are denoted by arrows. Despite severe multivessel CAD, the patient's global longitudinal strain (GLS) on cardiac magnetic resonance (CMR) was preserved (D). In contrast, the patient without obstructive CAD had poor GLS secondary to severe AS (I). Late-gadolinium enhancement on CMR demonstrates typical AS septal myocardial fibrosis and a small ischemic scar due to right coronary artery (RCA) stenosis (E, asterisk). Myocardial fibrosis due to severe AS (J, asterisk).

In this prospective cohort study, 98 patients were referred to TAVI due to severe AS between October 2018 and February 2022. Severe AS was defined by echocardiography Doppler assessment as a peak transvalvular flow velocity ≥4.0 m/s, and a mean pressure gradient ≥40 mmHg or AV area ≤1.0 cm^2^. All 98 patients underwent transthoracic echocardiography, CMR, TAVI computed tomography (CT), and invasive coronary angiography (ICA) before AV intervention. The average time gap between TAVI CT and preoperative CMR imaging was 60 ± 125 days. Exclusion criteria included other significant valvular diseases and inability to undergo CMR. Three patients had prior prosthetic AVs and were excluded. One patient was unable to endure the CMR examination and was therefore excluded. Patient selection is depicted in *Figure 1*. Patient characteristics are summarized in *Table 1*. An example of multimodality imaging in our study is shown in *Figure 2*.

**Table 1:** Baseline patient characteristics in patients with severe aortic stenosis

Characteristic	*N*	No CAD *N* = 74	CAD *N* = 20	*P*-value
*Demographics*				
Female	94	36 (49%)	5 (25%)	0.052
Age (years)	94	80 ± 7	82 ± 6	0.15
Body mass index (kg/m^2^)	94	27.0 ± 4.2	26.6 ± 4.9	0.70
Hypertension	94	65 (88%)	19 (95%)	0.43
Dyslipidaemia	94	58 (78%)	18 (90%)	0.28
Diabetes	94	14 (19%)	6 (30%)	0.99
Current smoker	90	26 (35%)	9 (45%)	0.42
GFR (mL/min)	93	68 ± 19	64 ± 19	0.46
NT-proBNP (ng/L)	43	923 (311–2056)	1028 (71–4359)	0.89
*Symptoms at presentation*				
Chest pain	94	27 (37%)	7 (35%)	0.87
Dyspnoea	91	65 (90%)	16 (84%)	0.45
Syncope	91	9 (13%)	0 (0%)	0.10
*Medications*				
ACEi or ATR II blocker	94	50 (67%)	12 (60%)	0.53
Beta-blocker	94	35 (47%)	9 (45%)	0.86
Statin use	94	41 (55%)	12 (60%)	0.71
*Coronary artery disease*				
Left main	94	—	4 (4%)	—
Left anterior descending	94	—	13 (14%)	—
Left circumflex	94	—	13 (14%)	—
Right coronary artery	94	—	9 (10%)	—
Multi-vessel disease	94	—	11 (12%)	—
Previous PCI	94	—	6 (7%)	—
Previous CABG	94	—	2 (2%)	—

Data are presented as number (percentage), mean ± standard deviation, or median (interquartile range).

ACEi, angiotensin converting enzyme inhibitor; ATR II, angiotensin II receptor blocker; CABG, coronary artery bypass graft; GFR, glomerular filtration rate; NT-ProBNP, *N*-terminal pro-B-type natriuretic peptide; PCI, percutaneous coronary intervention.

### CMR imaging and analysis

All the patients underwent preoperative CMR imaging as previously described.^11^ In brief, all the patients were imaged using the same 1.5 T system (MAGNETOM AvantoFit, Siemens Healthineers, Erlangen, Germany). The scan protocol included standard short-axis cine imaging from which left and right ventricular volumes and LV mass were obtained. Typical temporal resolution was 31 ms. Papillary muscles were included in the blood pool. Ventricular volumes and LV mass were normalized to body surface area. Ischaemic myocardial scar was defined as subendocardial or transmural late-gadolinium enhancement, which was obtained 10 min after an intravenous injection of 0.15 mmol/kg of gadoterate meglumine (Dotarem) using a phase-sensitive inversion-recovery technique. AV area was quantified by planimetry from balanced steady-state free precession cine images. Feature tracking of endocardial two-, three-, and four-chamber long-axis cine images were analysed by the auto-contour tool and manually corrected if necessary to obtain GLS by a single reader experienced with the GLS analysis (V.U.). One patient lacked a complete set of LV cine images and did not undergo GLS analysis. Both endo- and epicardial contours were drawn, and peak GLS was measured as shown in *Figure 2*. Medis Suite Qmass 8.1 software (Medis Medical Imaging Systems B.V., Leiden, The Netherlands) was used for all CMR image analysis.

Intra- and inter-observer variability in GLS was assessed in 15 CMR scans, with measurements blinded. GLS showed good intra-observer agreement (intraclass correlation coefficient [ICC] 0.88, confidence interval [CI] 0.69–0.96, *P* < 0.001) and moderate inter-observer variability (ICC 0.71, CI 0.28–0.89, *P* < 0.001).

### Cardiac computed tomography imaging and analysis

All patients underwent preoperative TAVI CT imaging, including standard non-contrast coronary calcium scoring (CCS), coronary computed tomography angiography (CCTA), and aortic CTA. Four patients were scanned using a 128-slice Siemens SOMATOM Definition Edge, 87 patients with a 256-slice Siemens SOMATOM Definition Flash, and three patients with a Siemens SOMATOM Force (all CT systems from Siemens Healthineers, Erlangen, Germany).

The standard coronary Agatston calcium score was re-measured for the study by an experienced cardiac imaging expert (V.U.). Coronary stents were identified using CCTA images and excluded from the CCS analysis. Coronary artery stenosis severity was obtained from the preoperative clinical ICA imaging reports. Obstructive CAD was defined anatomically as >50% stenosis in the left main coronary artery and ≥70% stenosis on coronary branches, or positive proof of ischaemia in an intermediate coronary lesion (30–70%) by nuclear imaging or invasive fractional flow reserve (FFR). Further imaging of intermediate stenoses was assessed by a clinical cardiologist as deemed necessary. Similarly, decisions of revascularization were made as part of clinical cardiology care. Patients with previous revascularization were not classified as obstructive CAD if the ICA at the time of AS treatment was non-obstructive. The time intervals from CMR to CT and from CMR to ICA were 60 ± 124 and 48 ± 94 days, respectively. Coronary CT analyses were performed using Syngo.via (Siemens Healthineers, Erlangen, Germany).

Intra- and inter-observer variability for AV calcium score was assessed in 15 CT scans. There was high intra- and inter-rater agreement (ICC 0.994, CI 0.982–0.998, *P* < 0.001, and ICC 0.990, CI 0.963–0.997, *P* < 0.001, respectively).

### Statistical analyses

Continuous variables are represented as a mean and standard deviation or median and interquartile range (IQR). Categorical variables are presented as numbers and percentages. Missing variables were considered random incidents. Correlations between parametric variables were analysed with Pearson correlation and non-parametric variables with Spearman correlation. In parametric continuous variables, a two-sample *t*-test or one-way analysis of variance was used. In nonparametric continuous variables, the Mann–Whitney *U* test or the Kruskal–Wallis test was used as appropriate. The chi-squared test of independence was used for categorical variables. The effect of categorical and continuous variables on GLS value was analysed with a general linear model. Statistical significance was set at *P* < 0.05. Statistical analyses were performed using SPSS (version 29.0. Statistical Package for the Social Sciences, International Business Machines, Inc., Armonk, NY).

## Results

### Prevalence of CAD

The final study population consisted of 94 patients. The prevalence of obstructive CAD on ICA was 20 (21%) patients, and 11 (12%) patients had multi-vessel disease. Previous revascularization was done in eight patients, of which six (6%) were percutaneous coronary interventions (PCI) and two (2%) were coronary bypass surgeries. The median CCS was 802 (IQR 302–2130). There was no statistically significant association between New York Heart Association class and CAD (*P* = 0.30). Similarly, CAD did not influence the occurrence of chest pain, dyspnoea, or syncope (*P* = 0.87, *P* = 0.45, and *P* = 0.10, respectively). Nt-pro-BNP was not elevated in patients with CAD (1028 (71–8180) vs. 923 (91–6783), *P* = 0.89). AV velocity by CMR was similar in AS patients with and without CAD (*Table 2*).

**Table 2:** Results of multimodality cardiac imaging in patients with severe aortic stenosis and with and without obstructive coronary artery disease (CAD)

Variable	CAD− *N* = 73	CAD+*N* = 20	*P*-value
**CCTA**			
Aortic valve calcium score	3004 (1768–4162)	2607 (2033–3954)	0.74
Coronary calcium score	527 (190–1520)	2236 (1132–3627)	<0.001
**Atherosclerotic burden**			
No atherosclerosis	5 (7%)	0	0.24
Mild (CCS <100)	8 (11%)	0	0.13
Moderate (CCS 100–399)	20 (27%)	1 (5%)	0.04
Severe (CCS 400–1000)	17 (23%)	2 (11%)	0.22
Very severe (CCS >1000)	23 (32%)	16 (84%)	<0.001
**CMR**			
AV area (cm^2^)	0.70 (0.50–0.70)	0.70 (0.50–0.90)	0.23
AV maximum velocity (m/s)	3.95 ± 0.96	3.73 ± 0.69	0.35
LV end-diastolic volume (mL/m^2^)	83 ± 26	90 ± 27	0.62
LV ejection fraction (%)	60 ± 9	58 ± 11	0.40
RV end-diastolic volume (mL/m^2^)	69 ± 18	74 ± 18	0.31
RV ejection fraction (%)	62 ± 9	63 ± 8	0.70
LV mass (g/m^2^)	67 ± 19	74 ± 19	0.29
Global longitudinal strain (%)	24 ± 6	24 ± 6	0.83
Ischaemic scar	7 (10%)	5 (25%)	0.07

Data are presented as a number (percentage), mean ± standard deviation, or median (interquartile range).

### Significance of CAD on myocardial contraction in AS

There was no difference between left and right ventricular EF in patients with and without CAD (*Table 2*). The mean GLS in patients with severe AS was 24.0 ± 6.1%. GLS was not reduced in patients with obstructive CAD compared to other patients (23.7 ± 5.9% vs. 24.1 ± 6.2%, *P* = 0.83). Patients with multi-vessel disease also had similar GLS compared to other AS patients (22.2 ± 6.1% vs. 24.2 ± 6.1%, *P* = 0.32). Previous revascularization was not related to lower GLS (22.2 ± 5.7% vs. 24.1 ± 6.2%, *P* = 0.46). Patients with an ischaemic scar on CMR had lower GLS (20.3 ± 5.1% vs. 24.6 ± 6.1%, *P* = 0.04) but not significantly lower LVEF (57.0% (51.8–60.3%) vs. 61.3% (55.5–66.4%), *P* = 0.05). The presenting symptoms of chest pain, dyspnoea, or syncope were not related to GLS (24.0 ± 6.7% vs. 24.0 ± 5.8%, *P* = 0.96; 24.0 ± 6.1% vs. 23.1 ± 6.9%, *P* = 0.66, and 21.6 ± 5.7% vs. 24.1 ± 6.2%, *P* = 0.25, respectively). CCTA and CMR characteristics of patients with and without obstructive CAD are depicted in *Table 2*.

**Figure 3:**
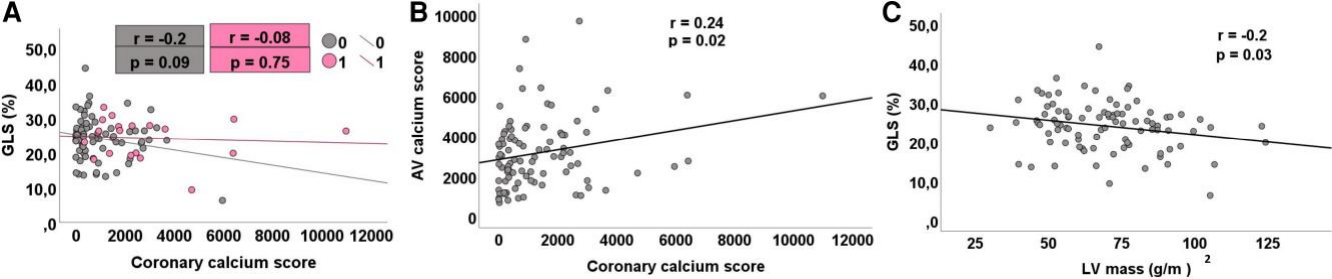
The relationship between coronary atherosclerosis, measured as the coronary calcium score (CCS), and myocardial contraction, aortic valve calcification, and left ventricular mass. There was no significant negative correlation between CCS and global longitudinal strain (GLS) when divided into subgroups according to obstructive (red) and non-obstructive (gray) coronary artery disease (*A*). There was a significant positive correlation between CCS and aortic valve (AV) calcium score (*B*), and a significant negative correlation between GLS and indexed left ventricular (LV) mass (*C*).

**Figure 4:**
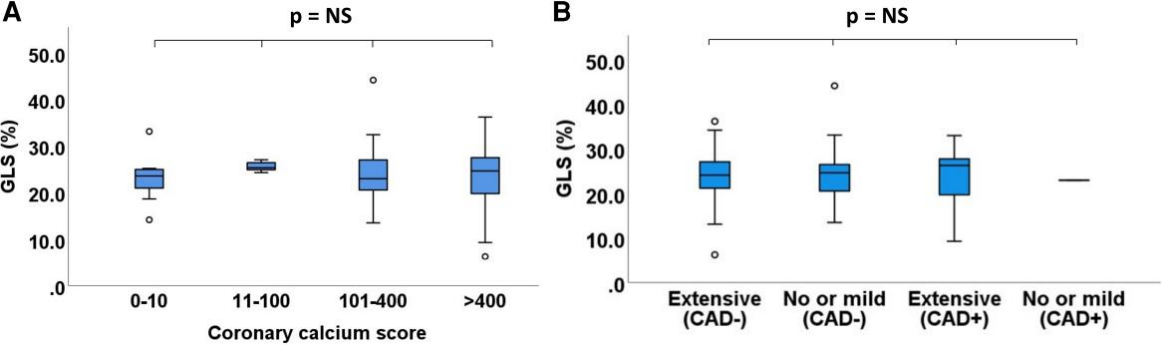
There was no significant difference between the extent of coronary atherosclerosis by coronary calcium score (CCS) and global longitudinal strain (GLS) (*A*). Four subgroups of patients stratified by the extent of coronary atherosclerosis (CCS >400) and obstructive disease, based on invasive coronary angiography results, did not differ significantly in GLS (*B*).

The association between GLS and the coronary calcium score is shown in *Figures 3* and *4*. In brief, the higher atherosclerotic burden measured by the coronary calcium score was unrelated to GLS.

### Aortic valve stenosis severity, LV hypertrophy, and myocardial contraction

The AV calcium score correlated with the coronary artery calcium score (*Figure 3*). The AV area, as measured by CMR, did not predict a decrease in GLS. Indexed LV mass was independently related to GLS (*Figure 3*). Patients with chest pain, dyspnoea, or syncope had comparable LV mass to other patients (70 ± 20 g/m^2^ vs. 69 ± 18 g/m^2^, *P* = 0.80; 70 ± 19 g/m^2^ vs. 72 ± 11 g/m^2^, *P* = 0.72, and 71 ± 25 g/m^2^ vs. 70 ± 18 g/m^2^, *P* = 0.92, respectively).

In the univariate general linear model (*Table 3*), women had higher GLS values (*P* = 0.046). Higher AV peak systolic flow correlated with better GLS values (*P* = 0.045). Higher indexed LV mass values, in turn, had a negative correlation to GLS (*P* = 0.013). When studied with a multivariate general linear model (*Table 4*), correlations with peak systolic flow and indexed LV mass remained statistically significant (*P* = 0.009 and *P* = 0.013, respectively).

**Table 3:** Clinical and imaging univariable predictors of myocardial contraction by global longitudinal strain in patients with severe aortic stenosis.

Predictive factor	Univariable	
	B (95% CI)	P
Female sex	2.54 (0.05; 5.04)	0.046
Age (years)	0.11 (−0.08; 0.30)	0.26
Aortic valve peak flow (m/s)*	1.42 (0.03; 2.83)	0.045
Aortic valve area (cm^2^)**	3.21 (−3.98; 10.39)	0.38
Obstructive CAD	−0.33 (−3.41; 2.76)	0.83
CCS (per 100 units)	−0.044 (−0.12; 0.029)	0.24
Hypertension	2.17 (−2.10; 6.43)	0.32
Diabetes	0.12 (−2.65; 2.88)	0.93
Dyslipidemia	−1.93 (−5,18; 1.33)	0.24
Ischemic scar	−4.3 (−8.0; −0.58)	0.02
LV mass (g/m^2^)**	−0.07 (−0.14; −0.07)	0.03

*by echocardiography; **by cardiac magnetic resonance imaging. CAD=coronary artery disease; CCS=coronary calcium score; CI=confidence interval; LV=left ventricular.

**Table 4:** Multivariable analysis of clinical and imaging predictors of myocardial contraction by global longitudinal strain in patients with severe aortic stenosis

Predictive factor	Model 1		Model 2	
	B (95% CI)	*P*	B (95% CI)	*P*
Female sex	2.54 (0.05; 5.04)	0.11	1.71 (−1.03; 4.13)	0.19
Aortic valve peak flow (m/s)^a^	1.42 (0.03; 2.83)	0.04	1.93 (0.52; 3.34)	<0.01
Ischaemic scar	−4.3 (−8.0; −0.58)	0.05	—	—
LV mass (g/m^2^)^b^	—	—	−0.09 (−0.16; −0.01)	0.02

^a^By echocardiography.

^b^By cardiac magnetic resonance imaging.

The use of an angiotensin converting enzyme inhibitor or an angiotensin II receptor blocker was not associated with higher GLS (24.6 ± 6.3% vs. 22.8 ± 5.8%, *P* = 0.20). Similarly, a beta blocker was not associated with better GLS (24.0 ± 6.7% vs. 24.0 ± 6.7%, *P* = 0.99). Statin use was unrelated to GLS (21.7 ± 5.9% vs. 21.7 ± 5.9%, *P* = 0.11), and also in the CAD subgroup (21.9 ± 6.8% vs. 26.0%, *P* = 0.13).

## Discussion

GLS has been a promising imaging marker for risk stratification of AS patients prior to valvular intervention.^12–14^ In this prospective multimodality imaging study, we examined the impact of obstructive CAD and coronary atherosclerotic burden on systolic function in patients with severe AS who underwent feature tracking GLS by CMR. In our study, myocardial remodelling, as assessed by LV mass, was associated with impaired GLS. Higher peak AV flow was associated with better GLS, which is logical, as myocardial contraction produces the AV gradient, and thus poorer contraction would result in lower AV velocity. In an extreme scenario, poor contraction may result in low-flow, low-gradient AS.^1^ To our surprise, obstructive CAD on ICA or diffuse atherosclerosis on CCTA did not result in lower LV GLS. This suggests that, usually, the severe AV stenosis and its secondary LV remodelling are more significant contributors to decreased GLS in severe AS patients than epicardial coronary artery obstruction. Indeed, we studied only patients with well-documented severe AS, of whom many were already symptomatic. Myocardial ischaemia might be a more important contributor to subclinical LV function in less severe AS. Moreover, GLS was measured at rest, whereas chronic myocardial ischaemia might be more important for cardiac contractility during exercise. However, an ischaemic scar was related to lower GLS. Our results suggest that intrinsic myocardial disease, rather than concomitant anatomical CAD alone, usually accounts for reduced GLS in this population. It should also be noted that ischaemic injury does not always necessitate obstructive coronary disease, and coronary stenosis does not always result in ischaemia.

### Interplay between myocardial ischaemia and AS

Chest pain is a common presentation of severe AS and AS may result in low myocardial perfusion by itself.^15,16^ Moreover, the hypertrophic remodelling and increased work due to high afterload also elevate myocardial oxygen demand. Arterial adverse structural changes due to perivascular fibrosis and capillary rarefication, elevated LV diastolic pressure compressing the endocardium, reduced diastolic filling time, and subsequent lower coronary pressure have been proposed as contributing factors to reduced myocardial blood flow in severe AS.^17,18^

### Clinical significance of CAD in AS

The presence of untreated obstructive CAD at the time of surgical AV replacement has been independently associated with adverse outcomes.^19^ In our study, patients were stratified into the CAD group based on the guidelines.^20^ However, the role of PCI in transcatheter AV replacement candidates remains uncertain, particularly following conflicting results from the ACTIVATION trial.^21^ Myocardial ischaemia may result from non-obstructive mechanisms such as increased LV afterload, hypertrophy, valvular outflow obstruction, and microvascular remodelling, which might attenuate the impact of epicardial coronary revascularization in the AS population. Myocardial infarction is associated with a worse outcome in the AS patients.^22,23^ Indeed, ischaemic scar was related to poorer GLS in our study.

### Limitations

Our population consisted of patients with only severe AS, and the number of CAD patients was limited and will not reflect the full spectrum of ischaemic heart disease. Therefore, we could not assess the impact of CAD on systolic function in patients with mild or moderate AS. Similarly, severe ischaemic disease due to chronic total coronary obstruction might have a greater impact on resting GLS than our study suggested. In addition, patients with previous myocardial infarction or ischaemic cardiomyopathy referred to valve intervention probably have poorer GLS than our unselected AS TAVI patients. Our study was not adequately powered or planned to assess the impact of CAD or feature-tracking GLS on clinical outcomes in severe AS. Intermediate coronary stenoses were further assessed by FFR or functional imaging by a clinical cardiologist, not systemically, as part of our study protocol.

The GLS was performed retrospectively for research purposes only and did not affect the clinical decision-making process at the time of the valve intervention. We did not measure regional strain values on different coronary regions, which might have been more sensitive to detecting the effects of one-vessel ischaemia on longitudinal contraction than global GLS, since regional strain analysis is less validated.^24^ We used endocardial feature-tracking GLS, which yields higher values than mid-myocardial strain, because we thought it might better track hypertrophic myocardium and endocardial injury in AS. The GLS results between CMR software packages are not interchangeable, which might affect the generalizability of the results. Moreover, our study selection criterion was the availability of TAVI CT, which excluded many younger patients referred for valve replacement surgery. In our older patient population, the interplay between dyspnoea, chest pain, and syncope with CAD and severe AS is complex and might be contributed to by other multi-morbidity. The study was a single-centre study, but Helsinki University Hospital is the sole aortic intervention referral centre in southern Finland serving 2.2 million residents in its collaborative area (about 39% of the Finnish population). Most of our patients were white Caucasian patients. Our CMR protocol did not include stress perfusion imaging. Therefore, we were unable to evaluate the relationship between myocardial blood flow and longitudinal strain in AS. We did not study LV relaxation, which might be worse in chronically ischaemic myocardium. We used the calcium score as a measure of plaque burden, which might have been more comprehensively characterized by novel CTA plaque burden software incorporating soft plaques into the analysis.

## Conclusions

Severe coronary atherosclerosis and obstructive CAD are common in severe AS. GLS correlated with LVH and the presence of ischaemic scar, suggesting that intrinsic myocardial disease, rather than concomitant CAD alone, accounts for reduced GLS in this population.

## Data Availability

The data underlying this article will be shared at reasonable request to the corresponding author.

## Lead author biography

Reetta Hälvä, MD, is a certified radiologist at Helsinki University Hospital, Helsinki, Finland. In addition to her clinical duties, she is engaged in cardiac imaging research at the University of Helsinki cardiac radiology department as a PhD student, with a particular focus on aortic stenosis and new cardiac magnetic resonance imaging methods, including 4D flow and speckle tracking.
